# Supplementary material for: Human Allogeneic Liver-Derived Progenitor Cells Significantly Improve NAFLD Activity Score and Fibrosis in Late-Stage NASH Animal Model
Source: Cells. 2022 Sep 13;11(18):2854. doi: 10.3390/cells11182854 (PMC9497074; doi:10.3390/cells11182854)
Supplement: Supplementary file 1 [file cells-11-02854-s001.zip › cells-1815650-supplementary.pdf]

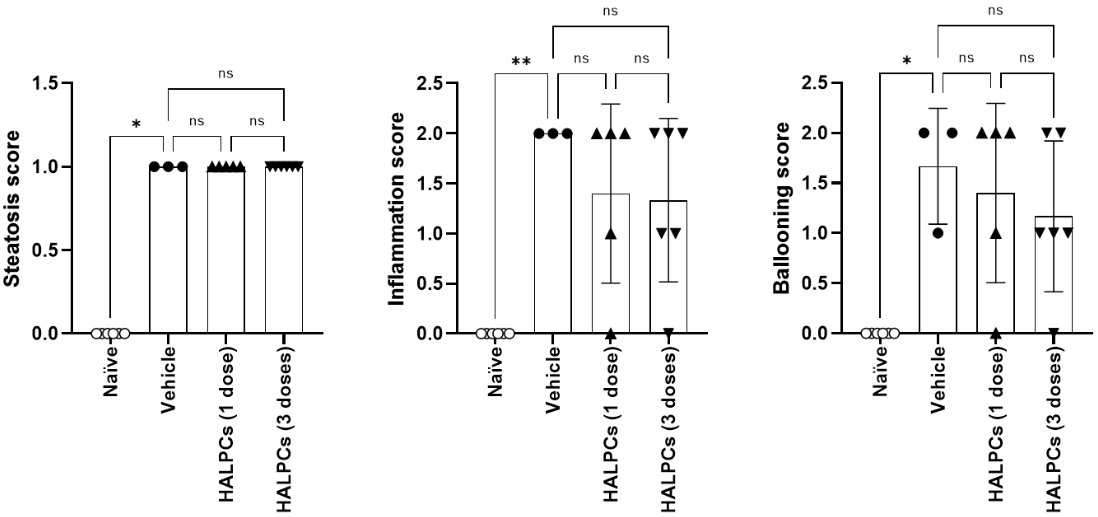

Supplementary Figure S1A. Early-stage NASH without immunosuppression. Statistical analyses were performed using Kruskal-Wallis (steatosis) or Tukey (inflammation and ballooning) Multiple Comparison Test on GraphPad Prism 9 (GraphPad Software LLC, San Diego, CA, USA). HALPCs: human allogeneic liver-derived progenitor cells; ns: not significant; *: $p < 0.05$; **: $p < 0.01$.

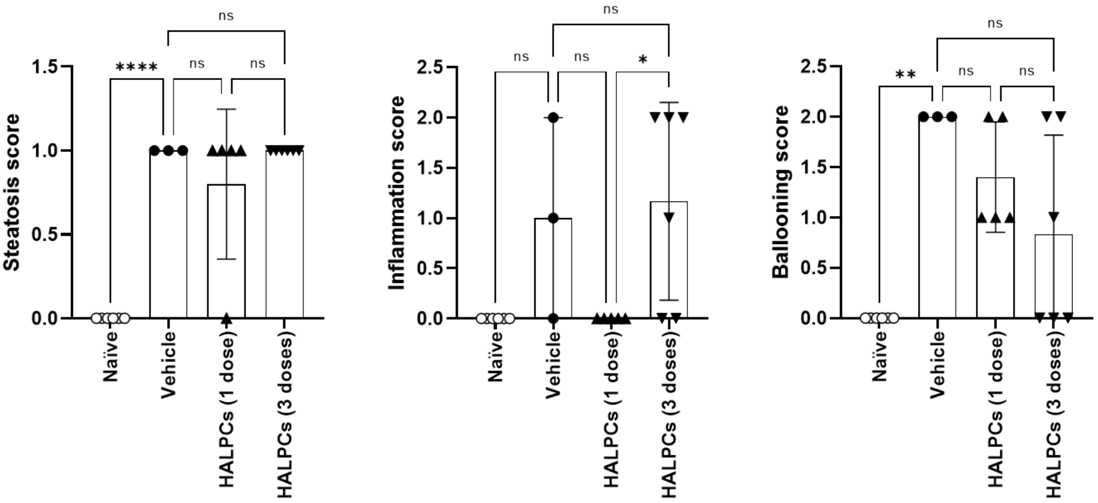

Supplementary Figure S1B. Early-stage NASH with immunosuppression. Statistical analyses were performed using Tukey Multiple Comparison Test on GraphPad Prism 9 (GraphPad Software LLC, San Diego, CA, USA). HALPCs: human allogeneic liver-derived progenitor cells; ns: not significant; *: $p < 0.05$; **: $p < 0.01$; ****: $p < 0.0001$.

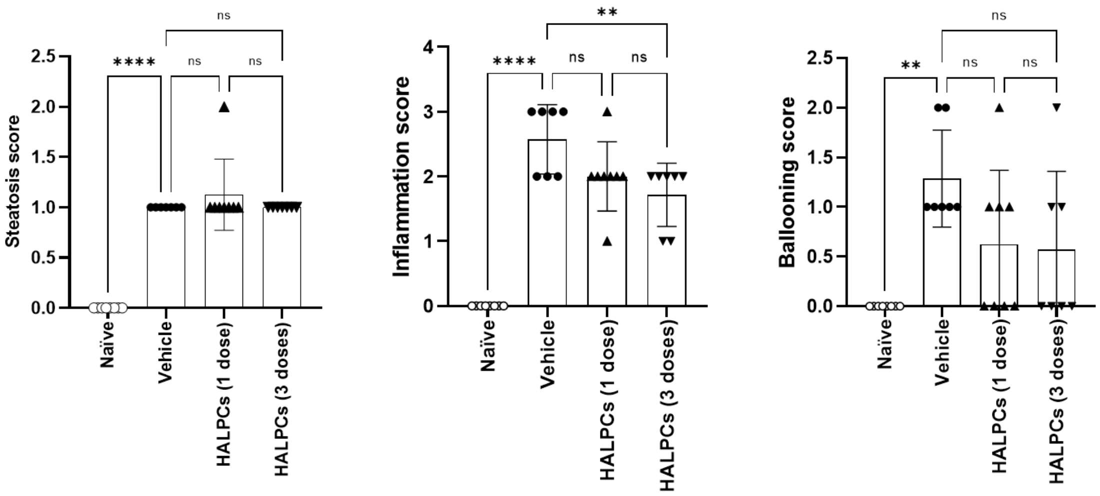

Supplementary Figure S2. Late-stage NASH with immunosuppression. Statistical analyses were performed using Tukey Multiple Comparison Test on GraphPad Prism 9 (GraphPad Software LLC, San Diego, CA, USA). HALPCs: human allogeneic liver-derived progenitor cells; ns: not significant; **: $p < 0.01$; ****: $p < 0.0001$.

Supplementary Table S1. Mean gene expression levels* in STAM mouse model (± SD).

	**Normal**	**Vehicle**		**HALPCs**			
				**2.5 × 10⁶ cells/mL**		**3 × 2.5 × 10⁶ cells/mL**	
**Immunosuppression**	−	−	+	−	+	−	+
**n**	**6**	**3**	**3**	**5**	**5**	**6**	**6**
**TNF-α mRNA**	1.0 ± 0.3	3.2 ± 1.3	1.8 ± 0.3	3.1 ± 0.7	2.9 ± 0.7	2.1 ± 0.9	2.7 ± 1.2
**α-SMA mRNA**	1.0 ± 0.7	1.6 ± 0.5	1.1 ± 0.3	2.5 ± 1.4	2.2 ± 1.0	1.4 ± 0.3	1.7 ± 0.8
**MCP-1 mRNA**	1.0 ± 0.7	4.9 ± 3.0	2.5 ± 0.9	9.9 ± 6.4	6.8 ± 4.9	2.5 ± 0.9	3.4 ± 1.7
**TMP-1 mRNA**	1.0 ± 1.0	3.8 ± 1.5	2.3 ± 0.2	7.2 ± 6.7	6.4 ± 6.1	2.9 ± 1.3	2.9 ± 1.2

* normalized to the level of reference gene 36B4 mRNA expression level.

HALPCs: human allogeneic liver-derived progenitor cells; n: number of samples; TNF: tumor necrosis factor; α-SMA: alpha smooth muscle actin; MCP-1: monocyte chemoattractant protein 1; TMP-1: Tissue inhibitor of metalloproteinase 1.

Supplementary Table S2. Mean body and liver weights in early- and late-stage NASH (± SD).

	Normal	Vehicle		HALPCs			
				**2.5 × 10^6 cells/mL**		**3 × 2.5 × 10^6 cells/mL**	
**Immunosuppression**	−	−	+	−	+	−	+
**Early-stage NASH (n)**	**6**	**3**	**3**	**5**	**5**	**6**	**6**
Body weight (g)	23.0 ± 1.5	20.5 ± 1.7	19.7 ± 0.5	20.8 ± 1.8	21.5 ± 2.1	19.5 ± 1.5	19.0 ± 1.6
Liver weight (mg)	970 ± 75	1484 ± 180	1362 ± 221	1468 ± 148	1686 ± 72	1326 ± 65	1351 ± 101
Liver to body weight ratio (%)	4.2 ± 0.3	7.3 ± 1.4	6.9 ± 1.0	7.1 ± 1.2	7.9 ± 0.7	6.8 ± 0.6	7.2 ± 0.7
**Late-stage NASH (n)**	8		7		8		7
Body weight (g)	29.1 ± 1.6		20.2 ± 2.0		20.2 ± 2.6		20.6 ± 3.3
Liver weight (mg)	1366 ± 74		1717 ± 103		1770 ± 276		1666 ± 308
Liver to body weight ratio (%)	4.7 ± 0.1		8.5 ± 0.5		8.8 ± 1.3		8.1 ± 1.2

HALPCs: human allogeneic liver-derived progenitor cells; n: number of samples.

Supplementary Table S3. Serum concentrations of biochemical parameters at Day 21 in early- and late-stage NASH (± SD).

	**Normal**	**Vehicle**		**HALPCs**			
				**2.5 × 10^6 cells/mL**		**3 × 2.5 × 10^6 cells/mL**	
**Immunosuppression**	−	−	+	−	+	−	+
**Early-stage NASH (n)**	**6**	**3**	**3**	**5**	**5**	**6**	**6**
ALT (U/L)	27 ± 4	72 ± 22	69 ± 43	126 ± 138	96 ± 65	41 ± 8	48 ± 13
AST (U/L)	98 ± 39	148 ± 48	200 ± 110	228 ± 229	188 ± 148	105 ± 23	103 ± 21
ALP (U/L)	444 ± 53	548 ± 88	614 ± 43	520 ± 290	619 ± 120	559 ± 91	454 ± 167
Triglycerides (mg/dL)	84 ± 24	475 ± 43	500 ± 0	350 ± 186	500 ± 0	437 ± 143	500 ± 0
Total cholesterol (mg/dL)	80 ± 9	170 ± 6	164 ± 24	156 ± 52	142 ± 20	170 ± 15	164 ± 27
**Late-stage NASH (n)**	**8**		**7**		**8**		**7**
ALT (U/L)	21 ± 3		65 ± 21		81 ± 42		65 ± 13
AST (U/L)	Not tested						
ALP (U/L)	297 ± 32		344 ± 74		339 ± 121		370 ± 87
Triglycerides (mg/dL)	147 ± 23		401 ± 86		413 ± 152		383 ± 146
Total cholesterol (mg/dL)	97 ± 7		165 ± 33		223 ± 100		190 ± 47

HALPCs: human allogeneic liver-derived progenitor cells; n: number of samples; ALP: alkaline phosphatase; ALT: alanine transaminase; AST: aspartate transaminase.
